# A Proof-of-Concept System Dynamics Simulation Model of the Development of Burnout and Recovery Using Retrospective Case Data

**DOI:** 10.3390/ijerph17165964

**Published:** 2020-08-17

**Authors:** Guido A. Veldhuis, Teun Sluijs, Marianne H. J. van Zwieten, Jildau Bouwman, Noortje M. Wiezer, Heleen M. Wortelboer

**Affiliations:** 1 Department Military Operations, The Netherlands Organization for Applied Scientific Research (TNO), 2509 JG The Hague, The Netherlands; guido.veldhuis@tno.nl; 2 Department Microbiology and Systems Biology, The Netherlands Organization for Applied Scientific Research (TNO), 3700 AJ Zeist, The Netherlands; teun.sluijs@tno.nl (T.S.); jildau.bouwman@tno.nl (J.B.); 3 Department Work, Health & Technology, The Netherlands Organization for Applied Scientific Research (TNO), 2301 DA Leiden, The Netherlands; marianne.vanzwieten@tno.nl (M.H.J.v.Z.); noortje.wiezer@tno.nl (N.M.W.)

**Keywords:** burnout, individual perceptions and behavior, social environment, scenario simulation, system dynamics

## Abstract

The phenomenon of burnout is a complex issue, which despite major efforts from researchers and organizations remains hard to prevent. The current literature highlights an increasing global prevalence of employees that are dealing with burnout. What has been largely missing is a more systemic, dynamic, and personal perspective on the interactions of the key determinants of burnout. Burnout can be seen as the outcome of a complex system involving feedback loops between individual mental models, individual behavior, and external social influences. Understanding the feedback loops involved may enable employees and organizations to intervene in burnout trajectories early and effectively. System dynamics (SD) modeling is a methodology that can describe the structure and behavior of a complex system. The current paper describes the development of an SD model of burnout. First, an expert- and literature-informed causal loop diagram (CLD) of burnout is developed. Then, a novel approach is developed to collect personal retrospective scenario data. Finally, the CLD and data are translated into a quantitative SD model. The potential of the SD model is illustrated by simulating the behavior of three realistic personas during the onset of and recovery from burnout. The process of development of an SD model of burnout is presented and the strengths and limitations of the approach are discussed.

## 1. Introduction

Prolonged work-related stress, together with associated negative health and performance, has become a societal concern around the globe affecting a high percentage of people and numerous organizations [1,2,3,4]. Work-related stress and burnout do not only have negative consequences for the health, safety, and wellbeing of workers, but also for the productivity and cost-effectiveness of the industries and services they work for [1,4,5]. In 2017, the World Health Organization proposed urgent actions to promote health by preventing mental health problems and related noncommunicable diseases that could occur later in life [6]. 

Globally, significant differences are noted between geographical regions, professions, and types of burnout measurement used. For example, for healthcare professionals the burnout symptom prevalence rates have been reported to be highest in sub-Saharan African and Asian regions, while European and Central Asian regions had the lowest rates [7,8]. In Europe, recent observations imply that on average around 10% of the EU+ workforce feels burned-out [9]. The total cost of lost productivity and social welfare associated with work-related psychosocial health problems in the EU were estimated at 617€ billion annually [10]. In addition, Western societies face an increasing burden of chronic stress-related health problems. Among these are cardiovascular diseases, type 2 diabetes, and mental disorders [3,6] Prolonged stress in relation to chronic overactivity or underactivity in cardiovascular and metabolic systems has been found to play a major role in development of cardiovascular disease and mental disorders [11,12,13]. As a consequence, chronic stress is an important risk factor for both mental and physical health effects later in life [5,14,15]. The involvement of environmental and epigenetic factors as risks for the onset of stress-related disorders is interesting, which may predispose certain individuals to stress-related health problems [16,17]. Recently, the WHO recognized workplace burnout in the International Classification of Diseases and Related Health Problems ICD-11 as a diagnosable condition (diagnostic code QD85), “resulting from chronic workplace stress” and encompassing a constellation of exhaustion, cynicism, and reduced efficacy [18]. However, instead of focusing on burnout as a disease, we advocate to conceptualize burnout as the outcome of a multidimensional complex problem deteriorating over time, and as such a process that may be reversed much earlier or treated more effectively when the complexity of the problem is better understood. 

Chronic work-related stress and burnout have been described extensively in the literature. In 1996, Maslach et al. defined burnout as a “syndrome characterized by emotional exhaustion, depersonalization, and reduced personal accomplishment caused by chronic occupational stress” [19]. Since then, several symptoms and influencing factors in various contexts were described [20,21,22,23,24,25]. This has led to theoretical models and extensions. Currently, a considerable research effort has been devoted to modeling the antecedents of burnout. One of the most widely used theoretical models of determinants leading towards burnout today is the extended job demands–resources model (JD-R) [23,26,27]. Extensive empirical support for the JD-R model has been gathered over the past decade in various countries [28]. The JD-R and other models of related concepts have uncovered many relevant antecedents of burnout and deepened our understanding of this phenomenon [20,28,29,30,31,32,33,34,35,36,37,38]. These theoretical models, however, are predominantly static and used to explain observations in cross-sectional studies. They do not, however, make the dynamic mechanisms explicit that explain the development of and recovery from burnout over time. Moreover, most of these studies have focused on only a few variables. Studies that did conduct longitudinal analysis did so for a limited number of time periods. The existing literature offers important pieces of the puzzle but seldom the full picture. 

**Figure 1:**
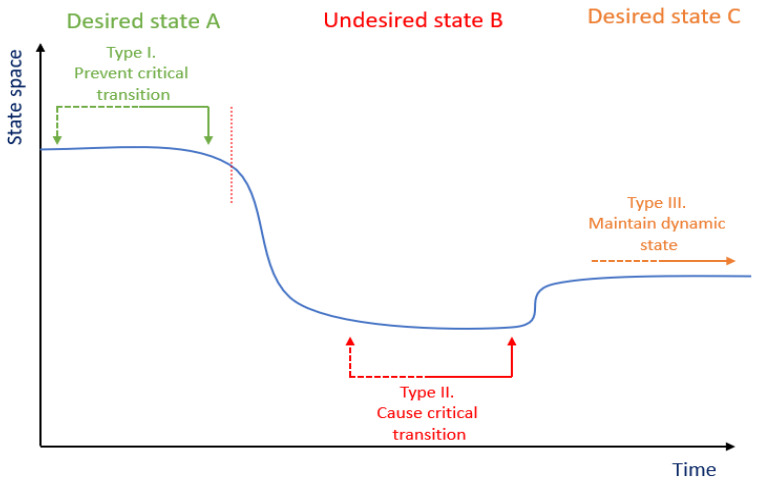
Illustration of complexity-science-based interventions. In terms of nonlinear dynamics, a system can move between multiple stable states as represented by the vertical axis. Type I interventions are targeted to prevent transition toward an unhealthy state, type II interventions are targeted to cause a transition toward a healthier state, and type III interventions are targeted to maintain a new dynamic healthy state. Adapted from Wietmarschen et al. [43].

What has been largely missing is a more systemic, dynamic, and personal perspective on the interactions of key determinants of burnout. Longitudinal studies have uncovered that the development of burnout displays a variety of patterns. Individual differences in onset and recovery of burnout seem to exist [39,40,41,42]. Such differences indicate the existence of a complex system that drives the behavior of the variables underlying burnout. Traditional cross-sectional methods are not suitable for describing the structure and dynamics of a complex system. Computational methods have shown potential for studying complex systems and are suitable for both describing the structure and behavior [43,44,45]. Longitudinal studies would be necessary to build and evaluate a data-driven computational model, but so far many studies have the limitation that a systemic dynamic vision of burnout has seldom been used to collect the appropriate data. Earlier, we illustrated our vision of complexity science providing methods to assess the essence of health-related issues and their dynamics, and to understand transitions in the system from one state to another state, for instance from a healthy state towards a disease state [43]. We argued that once the complexity and different states of the system are better understood, more complexity-science-based interventions and study designs could be developed to optimize intervention strategies (Figure 1).

In the present study, we were interested in whether the emergence of an unhealthy state of the individual, such as burnout, can be visualized and simulated using computational modeling, with the ultimate aim of using the model for an optimal and a more personalized intervention design. A similar approach provided encouraging results for obesity [43].

Multiple studies suggest that burnout is the result of a complex dynamic interaction of multiple variables from not only individual factors, but also social and workplace factors [5,24,46]. To reduce burnout, several integrated organizational intervention programs have been developed, yet the efficiency of these programs over a longer time was shown to be limited [47,48,49]. Recently, Pijpker et al. concluded from a literature review that involving employees in decision-making and enhance their job control and social support, while eliminating stressors, explains the effectiveness of combined interventions [50]. For a more sustainable solution of the phenomena, an integrated and personalized approach from a complex adaptive system view on health with engagement of stakeholders is, therefore, advocated [43,51].

In a complex adaptive system many interdependent factors, at multiple levels (e.g., chemical, individuals, organizations), continuously act and react with other factors. For example, in a biological ecosystem, factors that interact with each other (more heavy sunlight and a smaller water supply for example) can cause the ecosystem to deteriorate over time (for example, causing larger plants and larger animals to die, and allowing cactuses to grow). Intervening without a full understanding of the biological ecosystem, for example focusing solely on reduction of cactuses, and without the involvement of different stakeholders may show reduced efficiency and even worsen the situation.

In the current study, we considered how to combine existing knowledge and use computational modeling to describe the progression of burnout over time as a result of the underlying interactions. Our ultimate aim is to use the model to support both intervention programs and individual skills, as well as to optimize longitudinal study design to gain a holistic understanding of a client journey. The aim of this paper is to describe the applicability of a combined modeling approach to develop a simulation model of burnout development and recovery. The benefit of a computational model is that it not only simulates the dynamics of burnout over time, but that it also can provide a transparent depiction of the relationship between the underlying factors. As such, the model can function as an explanatory visualization of the complex relations between perceptions, behavior, and context that influence chronic stress, burnout, and recovery.

The subobjectives of the study were to: (1) explain the choice for system dynamics (SD) as the computational modeling method suited for this purpose; (2) unite expert, empirical, and theoretical inputs of burnout to capture a more holistic view of chronic stress, burnout, and recovery of burnout as experienced in the Western work environment; (3) capture longitudinal data on individual burnout experiences to develop and validate the burnout simulation model; and (4) integrate the collected patterns of behavior into a quantitative simulation model and study the potential of the model to simulate the trajectories of different personas on development and recovery from burnout.

## 2. Methods

In this section, we describe the steps in which the burnout model was developed. First, the choice for system dynamics (SD) as a computational modeling technique is explained. Second, the fundamentals of the method are described and illustrated via a simplified model on burnout. Then, the development of the SD burnout model is explained in three steps: (1) collecting key determinants of burnout and causal links via iteratively interviewing experts, with their information confirmed and supported by the literature and group model building sessions; (2) collecting retrospective personal perceived burnout scenarios using a novel experimental task, where participants are instructed to “draw their own dynamics”; and (3) integrating all collected information into a quantitative SD simulation model. Finally, simulation of three different personas was used to evaluate the potential of the model for visualizing the effects of personal perception and behavior on development of chronic stress, burnout, and recovery.

### 2.1. Choosing System Dynamics for Computational Modeling

The problem of burnout can be conceptualized as an undesirable state of a complex nonlinear dynamic system composed of physical, psychological and social variables. Feedback loops appear to be central to the problem. To capture the breadth of the system and its aggregated effects, together with its interactions, the choice for computational methodology has therefore been directed to system dynamics (SD) modeling. In contrast to other computational methodologies, SD is a computer modeling methodology that is used to represent and analyze both system structure and complex nonlinear behavior. The methodology focusses on generating insights, sharing awareness, and improving (system) performance, and is, therefore, a suitable fit for the purpose of this research. SD modeling can be used to test hypotheses on how variables interact by expressing the causal links between them using differential equations. It is often used to understand and simulate the behavior within a system in different states (e.g., burnout, not burned-out) and in different scenario’s (e.g., different personas). This is especially valuable for visualizing the effects of “what if” scenarios, which cannot be extracted using conventional empirical methods. SD has its intellectual origins in control theory, management science, and digital computing [52] as a method for helping managers better understand and control corporate systems. As of today, SD is applied to topics in a wide variety of academic disciplines, yet only to a limited extent in the preventive healthcare domain. The premise of the methodology fits well with the preventive healthcare domain, as an understanding and perception of your own health and behavior in relation to different contexts are key for a healthy lifestyle [53].

SD modeling has been used to visualize the dynamic behavior of systems consisting of multiple processes, either at the individual, group, or society level [43,44,45,54,55]. Applications of SD to stress and wellbeing appear to be limited. Homer (1985) was the first to describe burnout as a system of feedback relations at an individual level based upon his own experience (*n* = 1) [54]. Wu et al. used SD modeling to create awareness of the employee’s work–family tensions in the construction industry based on an extensive literature review [55]. Jetha et al. developed a multidimensional SD model of workplace stress among nursing aides and evaluated the model via a survey of 950 nurses collected biannually over one year [45]. This model included perceptions of organizational conditions (e.g., job demands and job control), workplace social support (i.e., managerial and coworker social support), workplace safety, and demands outside of work (i.e., work–family conflict). Jetha et al.’s SD model was able to depict the perceived importance of job control and job demands relating to long-term workplace stress. Model simulations showed that increasing job control and decreasing job demands led to a decline in workplace stress, while workplace social support, workplace safety, and work–family conflict appeared to be less influential within the broader system [45]. None of these simulation models, however, described the full complexity of burnout to understand the effect of personal perception and behavior on chronic stress, burnout, and recovery. Visualizing and discussing causal structures prior to a decision have been shown to aid understanding and confidence in changing ingrained patterns [56]. As individual differences in onset and recovery of burnout seem to exist [25,39,40,41,42], an understanding of the positive and negative feedback loops through visualization may open the door to a deeper understanding of the processes that lead to burnout and recovery. Therefore, to develop a SD model of a complex, personal, and multidimensional problem, such as burnout, theoretical models derived from experts of different disciplines, details confirmed by the literature, as well as participants’ personal perceptions of their journeys and behavior related to the development of chronic stress, burnout, and recovery, all need to be synthetized [57,58].

### 2.2. Describing the Fundamentals of SD Modelling Illustrated via a Simplified Model on Burnout

**Figure 2:**
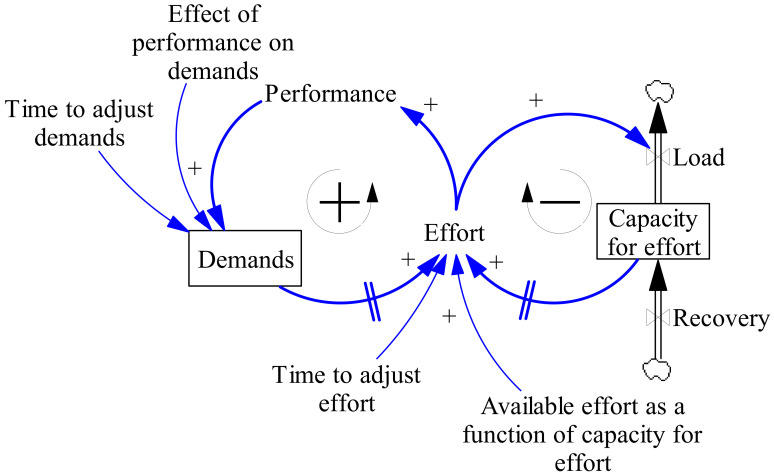
Example of a simplified system dynamics (SD) model of burnout to illustrate several key elements of a SD model. Stocks accumulate over time and are indicated by rectangles. Flows indicate the rate of change of a stock and are depicted as black arrows. Variables are connected with blue arrows indicating causal links, where a plus indicates that an increase (decrease) in the variable at the tail of the arrow constitutes an increase (decrease) in the variable at the head of the arrow, all things else being equal; whereas a minus indicates that an increase (decrease) in the variable at the tail constitutes a decrease (increase) in the variable at the head, all things else being equal. Delays (//) indicate that the increase occurs after or over multiple time periods. Feedback loops are indicated as black circular arrows. A feedback loop can be reinforcing (+) or balancing (−).

First, to illustrate the method used to develop the SD model, we will start by describing a simplified model of burnout, including some important properties of dynamic complex systems and their implementation (Figure 2). The system dynamics method essentially makes use of three constructs to capture these dynamics over time—stocks, flows, and variables, all represented in Figure 2. A stock, which is visually represented by a rectangle, represents a quantity with a specific condition in the system at a certain point in time (a state). A flow, indicated with a double-lined (black) arrow, represents the change in a stock at a time interval. The state of a stock depends on its inflow, outflow, and initial quantity. As such, flows are dependent on stocks and stocks are dependent on flows. Additional intermediate variables are often used to describe the system more clearly. These variables are indicated by words preceding single (blue) arrows. Variables are connected with blue arrows indicating causal links, where a plus indicates that an increase (decrease) in the variable at the tail of the arrow constitutes an increase (decrease) in the variable at the head of the arrow, all things else being equal; whereas a minus indicates that an increase (decrease) in the variable at the tail constitutes a decrease (increase) in the variable at the head, all things else being equal. Delays (//) indicate that the increase occurs after or over multiple time periods. The interactions between stocks drive the system behavior through positive or negative relationships that cause the accumulation or depletion of the stocks. This interaction often takes the form of a feedback loop, indicating a self-reinforcing (+) or balancing (−) effect. Feedback loops are visualized using a loop symbol. For example, in Figure 2, the more effort exerted, the better the performance, resulting in more demands. Over time, the person improves, increasing their effort and performance (a self-reinforcing loop). The increased effort, however, increases the load (outflow), draining the capacity for effort quickly if recovery (inflow) remains unchanged. With a lower capacity for effort, the effort itself decreases too (balancing loop). The interaction and feedback between multiple endogenous variables, stocks, and flows create the dynamics of the system over time. As such, endogenous variables affect and are affected by other system variables. Exogenous variables, such as an education program or intervention, are outside (input) variables that affect but are not affected by the system.

Next, we use Figure 2 to illustrate how SD quantifies the relationships between variables into equations, describing a change in a variable over a certain time. For example, the amount of effort an individual can invest (“capacity for effort”) is modelled as the variable “capacity for effort” (Equation (1)). The stock “capacity for effort” is an accumulating variable, of which the “*capacity for effort*” at a certain time (*t*) is mathematically modelled as an integral of its inflow (“*recovery*”) and outflow (“*load*”) over a period of time (“*ds*”) added to the target variable at the time before the change (*t_0_*).

(1) $$Capacity\;for\;effort\left(t\right)=Capacity\;for\;effort\left(t_{0}\right)+\overset{t}{\underset{t_{0}}{\int }}\left[Recovery-Load\right]ds$$

An individual, however, does not have an unlimited capacity for effort. This makes it important for individuals to manage the amount of effort they have available. As people work, they perceive “demands” (either private or work) and they will invest effort to meet the demands of their job. However, this effort will not exceed the demands that the person perceives nor the amount of effort they can invest. This creates a negative feedback loop (Equation (2)), whereby the person uses capacity for effort to invest effort and reduces their effort when their capacity for effort becomes too low (i.e., the person becomes exhausted).

(2) $$Effort=Min\left(Demands,\;f\left(Capacity\;for\;effort\right)\right)$$

**Figure 3:**
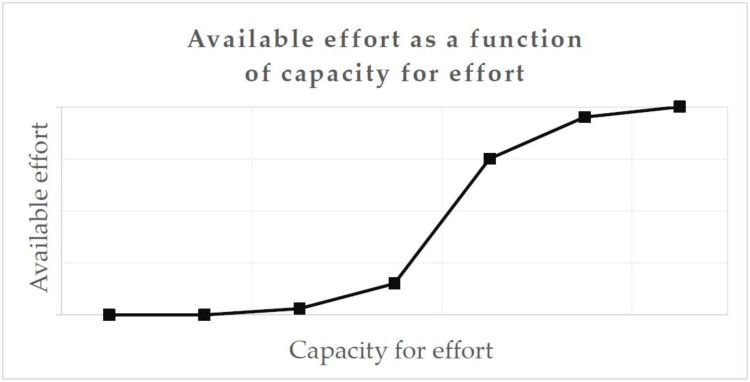
Example of a nonlinear function—available effort as a function of capacity for effort.

This, in general, is a nonlinear effect, illustrated in Figure 3. When “capacity for effort” is high, e.g., when the person feels well-rested, a small reduction of the “capacity for effort” might not restrain the “available effort” much. However, at some point, additional demands and fatigue might cause a sharp decline in the ability to invest effort.

For the people who perform well at their job, their performance increases over time. When performance grows, so do the expectations for future performance, thus increasing the demand. The variable “demands” is, therefore, modelled as a “goal-seeking” variable, a stock that gradually adjusts to a target (Equation (3)). In this case, the target is set by an extrapolation of the current performance.

(3) $$Demands\left(t\right)=\;Demands\left(t_{0}\right)\;+\;\int_{t_{0}}^{t}\left[\frac{\left(Performance*Effect\;of\;performance\;on\;demands\right)-Demands(t_{-1})}{Time\;to\;adjust\;demands}\right]dt$$

An increase in demands leads to an increase in effort, which leads to an increase in performance, and so on. This is referred to as “positive feedback”, a self-reinforcing process. Notice that both stocks make the future behavior of the system depend on its history. The effort a person has invested in the past has an influence on the effort they can invest in the future. The adjustment process used to model demands might also be used to include other delays. For example, a person might not be able to adjust the amount of effort devoted to their job instantaneously. When demands change, time might have passed before the change is observed and effort is adjusted accordingly. To simulate the adaptation process of the adjustment to the demand, within SD a “first-order smoothing” function is used. Such a function implies that the person in the current model gradually updates their perception of some variable (e.g., demands) by averaging past information. The endogenous properties described above can lead to undesirable behavior and lower performance. Initially, most persons have sufficient capacity for the effort, which allows the positive feedback loop to be dominant, leading to an increase in the amount of demands, and correspondingly the effort that is invested to meet these demands. 

**Figure 4:**
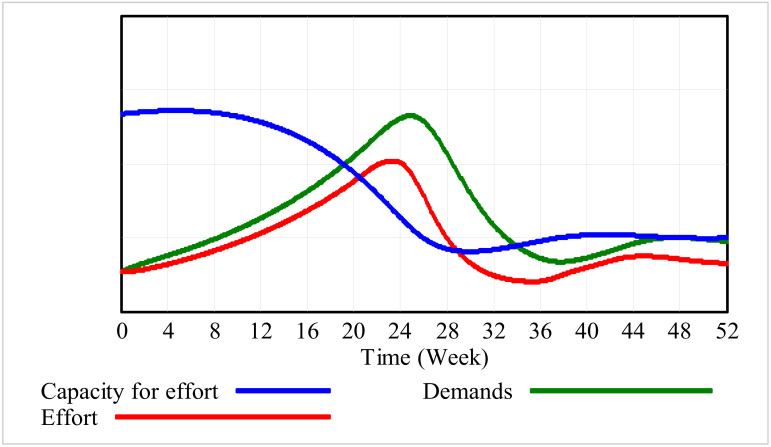
Example of simulation results of undesirable behavior leading to a lower amount of effort a person can invest. The y-axis indicates the perceived capacity of effort, perceived amount of demands, and perceived effort (low, mid, and high, quantified in the model as 0, 0.5, and 1, respectively).

An example scenario is depicted in Figure 4 for illustration. In this exemplary graph, a hypothetical person takes on a new challenge given by management, starting with enthusiasm and a high capacity for effort (blue line) for (a set of) new tasks (green line). However, when the amount of demands (green line) increases, the effort the person must deliver (red line) increases as well, to a point that the person becomes exhausted, reducing their capacity for effort. This is visualized by a tipping point in the red line after week 24. Due to exhaustion, the amount of effort the person can deliver reduces quickly. In this case, demands also reduce, as this hypothetical person struggles to meet the new demands. This gives this person some room to recuperate to such a point that the capacity for effort increases again. However, as the capacity for effort builds up again, so do the demands. Without dealing with the situation, finally around week 52, a stable (but often unwanted) equilibrium is reached—the capacity for effort that the person can invest (in his work or private situation) is much lower than what could potentially be invested if demands, effort, and recovery would have been well balanced from the beginning.

This small example of a SD model of burnout, however, does not capture the full complexity. 

### 2.3. Collecting Expert Information on Burnout to Build the SD Model

To collect all key determinants and causal links involved in the development of chronic stress, burnout, and recovery of burnout, as experienced in a European work environment, we started the research by iteratively interviewing five experts in psychology, systems biology, neurology, organizational psychology, and sociology. Experts were health researchers with research experience ranging from ten to thirty years and all experts had scientific track records related to chronic stress or burnout-related issues. All experts were living and working in the Netherlands. During this process, literature (focusing on the European work environment) was used to confirm and support the expert-informed determinants and their causal links.

Four Dutch experts of the research team participated in a total of five group model building (GMB) sessions. We followed the GMB approach as described by Vennix et al. [57]. Each of the sessions lasted 120–180 min. During these sessions, the experts discussed the information gathered from the interviews and literature and gradually built the model. This process was facilitated by a SD modeler. Discussions during the GMB and collaborative model formulation led to a deep inquiry into the properties of the system. VENSIM Simulation Software© developed by Ventana Systems (DSS version 7.0 (Ventana Systems, Inc., Harvard, MA, USA)) was used to record and visualize the progress in a causal loop diagram.

### 2.4. Collecting Retrospective Personal Perceived Burnout Scenarios

The conceptual model structure was expert- and literature-informed. To develop a quantitative model, empirical and qualitative scenario data are needed. As detailed longitudinal quantitative data of burnout appeared to be scarce, an experimental task was developed to collect the retrospective longitudinal data on individual burnout experiences. Participants were asked to provide data by sketching graphs-over-time. The data were validated through consensus by interviewing participants on details of their response. In this form of validation and model construction, experts decide on the model inputs with consensus from the participants. Participants were asked to describe their burnout experience by sketching the development over time on a template, following four questions. Each of the questions were explained and supported by examples in their questionnaire.

The sketch assignments were:

1. Identify the start and end date of your burnout experience on the x-axis, and include the following assignments in the timeline.
  - Identify developments in your professional life and mark during which time they were active;
  - Identify developments in your personal life and mark during which time they were active;
  - Sketch graphs of the following 7 variables as perceived by yourself during the timeframe: amount of demands, effort, perceived efficacy, appropriateness of resources, engagement, need for recovery, stress responses.
2. Interventions: Note down at which moments in time activities were conducted to improve your situation.

After the task, an in-depth interview by two project researchers with each individual lasting 60 min was held, in which participants were asked to explain what they had sketched. Thereafter, dynamic profiles of processes and relevant behavior involved for the burnout model were extracted from the sketches by the researchers. The concepts were discussed within the project team and further conceptualized for the system dynamics model.

Participants were recruited via an email to employees working in a project-driven research institute in the Netherlands. Inclusion criteria were development of chronic stress, burnout, and a full recovery experience while working at the same project-driven research institute in the Netherlands, aged between 25 and 60 years, either male or female. Participants who were interested in participating in this study were sent an invitation describing the purpose and outline of study. The study protocol was in accordance of the World Medical Association Declaration of Helsinki and approved by the Ethics Committee of TNO.

### 2.5. Integration of Collected Information into the SD Model and Studying the SD Model Behavior via Simulation Runs 

Lastly, the conceptual structure model plus collected personal scenario data were translated into a quantitative stock-and-flow SD model. The dynamics between the key model components that were indicated by the experts and confirmed by the literature were adapted in line with the insights that were obtained from the participant exercise. Resulting empirical variables following the exercise were integrated in the model, equations were specified, and multiple simulations were executed to iteratively improve the SD model. In the end, simulations were run with the final SD model to study the effect of three different personas on the development of chronic stress, burnout, and recovery to study the potential of the model.

## 3. Results

### 3.1. Expert-Informed Structure of the SD Burnout Model

The experts confirmed that one of the most widely used models today to research burnout is the extended job demands–resources model (JD-R) [23,26,27,28]. Beyond the JD-R model, experts also indicated that other models addressing related concepts have uncovered many relevant antecedents of burnout and deepened the understanding of the phenomenon [20,21,23,28,29,30,31,32,33,34,35,36,37,38]. The experts confirmed that a more holistic, systemic, and dynamic perspective of burnout is missing. Several studies have taken first steps in this direction, for example by identifying “positive gain spirals” [22,59,60,61,62], which in computational modeling are identified as “positive feedback loops” [52]. This can be described as the positive relationship between variables—if one variable increases or decreases (the “cause”), the other variable increases or decreases with it (the “effect”), causing the first variable to increase or decrease even more (the “feedback loop”). In Table 1, an overview is given of existing theoretical models that were selected to inform the SD model structure.

### 3.2. Retrospective Personal Perceived Burnout Scenarios

An experimental technique was developed to collect personal burnout and recovery experience data, where participants were asked to “quantify” and to “draw their own dynamics” for seven variables (i.e., amount of demands, effort, perceived efficacy, appropriateness of resources, engagement, need for recovery, stress responses) in relation to three timelines (interventions, developments in the work environment, developments in private life).

**Figure 5:**
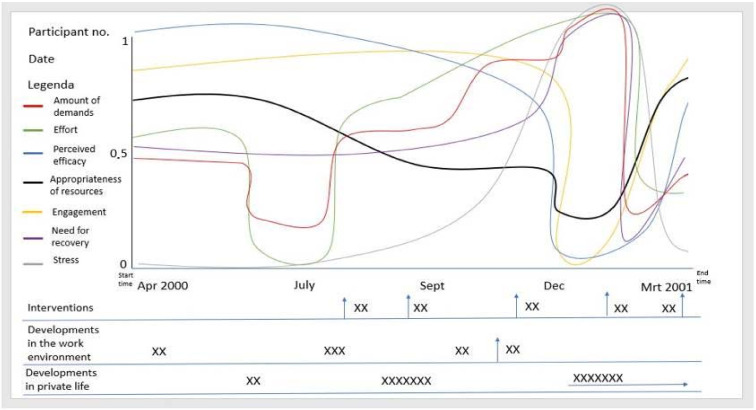
The response from one of the participants (digitalized with Microsoft PowerPoint). The top section shows the graphs of the seven perceived variables (amount of demands, effort, perceived efficacy, appropriateness of resources, engagement, need for recovery, stress responses) sketched by the participants. The bottom section shows the three timelines (from-top-to-bottom: interventions, developments in the work environment, developments in private life). The actual responses included notes on the developments.

In total, 8 participants (two females, 6 males, all scientific researchers of various expertise, aged between 30 and 60 years, with burnout and recovery experience) responded to the open invitation. They were informed on the purpose of the program in more detail and gave their informed consent for inclusion before they participated in the study. All participants gave permission to use their personal experience and insights for incorporation in the model. Seven out of eight participants completed the sketching task in between 30 and 60 min. One of the eight participants decided to quit the exercise during the process. All seven respondents indicated that the task was clear and that they were satisfied that their response to the exercise adequately captured their personal retrospective burnout experiences. They reported that the task was a very insightful way to understand and share their story, especially to have the opportunity to detail their experience in seven separate yet connected variables. In one out of the seven cases, the researchers were under the impression that the respondent misinterpreted some of the questions. In-depth interviews revealed that all other participants had correctly interpreted the definitions of the seven variables and timelines. All participants had described their burnout and recovery experience via sketch graphs of the seven variables as perceived by the participant during the timeframe. A typical sketch is presented in Figure 5.

The patterns of behavior for different phases relevant to the SD burnout model were strikingly similar for all participants. There were differences in the timeframes sketched, ranging from about 1 year to 3 years, with some exceptions where the respondents sketched 10 years, in which multiple episodes of burnout symptoms were visible. Three phases were broadly distinguished as the “build-up phase”, the “crash phase”, and the “recovery phase”, which are described below.

#### 3.2.1. Build-Up Phase

- Perceived increasing demands from low to medium to high levels. Demands were associated with experienced pressure, which had its origins in demands such as role unclarity, new and challenging responsibilities, and perceived expectations from supervisors;
- Perceived increasing effort from medium-high levels to high levels;
- A perceived need for recovery that persisted at relatively low levels for a prolonged time, after which the system showed a steep increase. Participants reported that they were unaware of their fatigue for some time. Some participants reported that in hindsight they showed symptoms of fatigue, such as a decrease in cognitive or emotional functions;
- Perceived resources were drawn as either being at a low level or decreasing to a low level. Participants reported that they had insufficient resources available or that they were unable to make use of the available resources.

#### 3.2.2. Crash Phase

- The crash phase showed great similarities between cases—a peak in perceived demands and effort followed by a sudden drop. This peak was accompanied by a peak in perceived stress response and a drop in perceived efficacy;
- The onset of the crash appeared to be triggered by one of two mechanisms, the first whereby the respondents commented that they reached a point at which further effort was impossible due to perceived exhaustion and reduced emotional and cognitive functioning. In the second case, participants reported to have invested excessive effort, yet did not perceive or receive expected rewards. They perceived to have received rather negative feedback from a client or supervisor. In the latter cases, the variable “engagement”—which is defined as a uniquely positive, fulfilling, work-related state of mind that is characterized by vigor, dedication, and absorption [65]—showed a significant drop and prolonged low values. These accounts of events led to more cynicism than the other cases.

#### 3.2.3. Recovery Phase

- All participants sketched a plateau of lowered (but not absent) activity, self-efficacy, and in some cases engagement. The timeframe of this plateau differed between 1 month and 1 year. The middle and end of the tableau sketch sections showed a recovery of self-efficacy, with lowered stress responses and need for recovery. After these sections, the plateau sketches showed a gradual return of demands and effort;
- Almost all participants commented in the interviews that they had learned a similar lesson from the burnout experience that led them to manage their professional life in a new way, adjusting their priorities for work and life goals and learning to better regulate demands (by saying “no”, delegating, and matching their role with their capabilities).

### 3.3. The SD Burnout Simulation Model

**Figure 6:**
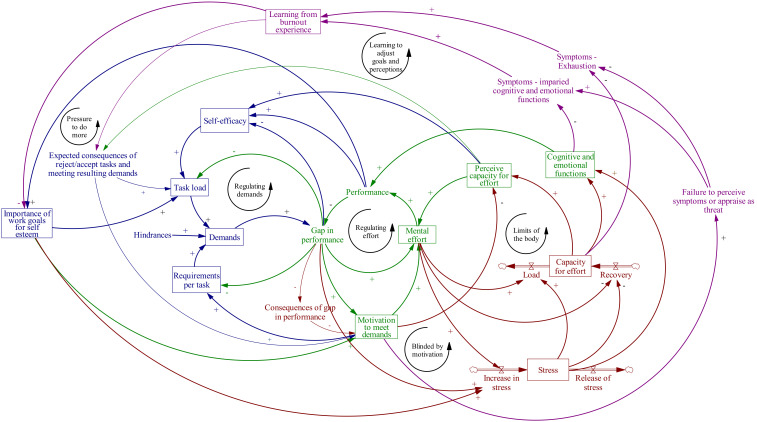
A system dynamics model of burnout integrating existing theoretical models, information from experts and literature, and personal burnout experiences. Colors indicate four main identified processes—regulating demands (blue), regulating effort (green), impacts on body and mind (red), and burnout symptoms (purple).

The collected personal scenario data and the expert-informed conceptual model were integrated into a quantitative SD model. Figure 6 presents the final SD burnout simulation model, mirroring the integrated information from experts, literature, and individuals with burnout experience regarding the effects of personal perceptions, characteristics, and behavioral changes on the development of chronic stress, burnout, and recovery. The SD model is built on four main identified processes—regulating demands, regulating effort, impacts on body and mind, and burnout symptoms. Each process within the SD model is pictured in a different color (Figure 6) and is described in more detail below.

#### 3.3.1. Regulating Demands

In the SD model, the “demands” stock consists of certain tasks (or a single particular task) and associated requirements, either at work or in a private situation. During work employees are confronted with requests to perform their daily tasks. A request can be an explicit question to perform a certain task, for example by a manager, a colleague, a client, or a private relative. However, a request can also be more implicit. For example, the employee might feel obligated to prepare for a meeting. Out of both the expert’s information and the participant experiment, a core variable arose to determine the demand load—the variable “task load”. How many requests the employee is confronted with, in other words how the “task load” of the individual looks, depends on the occupation of the individual and their performance until that point, the relationships of the individual with colleagues, the organizational culture, and the individual’s private situation.

**Example** **1.** *Tim has just started working at an organization. He performs very well. New projects keep coming his way. The projects become more complex and Tim gets more and more responsibilities. Tim is seen as having high potential. He gets extra assignments and is asked to join all kinds of programs and meetings. Tim has a hard time managing the “regulating demands” loop*.

Those requests to perform tasks are often accompanied with quality, quantity, or process requirements. Spoken and unspoken requirements can be laid down by the organization, a team, a manager, a colleague, or a private relative. The external requirements are perceived and interpreted by the employee in a certain way. For instance, if an organization or a manager provides requirements that are rather descriptive, there is much space for interpretation. How employees perceive and interpret the external requirements is determined by their personality, history, context, (learning) experiences, motivation, and own personal standards of quality. In the model (Figure 6), “demand” is, therefore, defined as a combination of “requirements per task” (+) multiplied by the perceived “task load: (+), with “hindrances” (+) as reinforcing factor.

**Example** **2.** *When Alexandra’s team manager asks her to write a specific report, her manager is—as always—unclear about the requirements for the form, content, and deadline. On the other hand, her manager is making her feel that this is an important task that must be done perfectly. In her team, she has noticed what can happen when one is not performing well or makes a mistake in this organization. Alexandra knows that the standards are high. Her parents and teachers encouraged her to be an excellent student. They rewarded her for getting good grades. The better she performed, the better she felt about herself. Good performance has brought her much. Alexandra aims high. She always did. Alexandra gets caught up in the “blinded by motivation” loop*.

A request to perform a task and the associated requirements are only considered a demand if employees feel they must act upon the request and its requirements. This feeling arises as a consequence of the employee having accepted a request to perform a task or at least not having rejected the request. Whether an employee accepts or denies a request depends on the expected consequences of accepting or rejecting and on the expected consequence of failing or succeeding in meeting the requirements of the task. Those expected consequences only matter if the employee attaches personal importance to those consequences. How important is work to the individual and their work performance in building up their self-esteem (the latter defined as a personal sense of self-worth)? The personality of the individual and their culture, world view, and experiences in the past play important roles in this process. Additionally, an employee has to deal with demands in relation to a specific work and private context. This context could contain hindrances, such as noise and role unclarity. This might put an extra burden on the demand.

**Example** **3.** *Karim oversees multiple projects at the same time. Now he is asked to be a project leader of a complex international project. He never worked on an international project before. He is filled with pride as they all have so much confidence in him. If he accepts the request, he satisfies his project leader. If he succeeds, he believes he will get an outstanding performance review. He thinks of all the recognition he will get when he succeeds. They will continue asking him for new projects. Nevertheless, he will certainly have to work overtime—something he has done for weeks now. Rejecting the request will bring him some relief and some time to recover from all the effort he has made lately. On the other hand, if Karim rejects the request, he thinks his project leader and some colleagues will persistently try to convince him to change his mind. They did so before. Karim believes it will have consequences for his performance review and his chances on=f getting a promotion. It seems like his self-esteem is tied to his success at work. His work gives him sense of purpose. Karim falls in the “pressure to do more” loop*.

#### 3.3.2. Regulating Effort

The second main process in the model (Figure 6) describes the regulation of effort by the employee to deal with the demands. Dealing with demands requires (mental) effort from the employee. How much effort employees initially invest depends mostly on their motivation to comply with the requirements of the demands at that moment. This motivation is based, among other factors, on their expectations of the consequences of failing or succeeding in meeting the requirements of the task. Ultimately, they are intrinsically motivated by their own appreciation of those consequences. How important is work to the individual and their work performance in maintaining or strengthening their self-esteem and wellbeing? How much effort employees will initially invest also depends on their (perceived) capacity for effort—fatigue and cynicism will reduce the effort that is invested, possibly ending in a negative spiral.

The (mental) effort employees invest will likely affect their performance. If employees perceive the performance as “sufficient to meet their requirements of the demands”, their self-efficacy will be boosted. If this performance does not comply with the requirements (yet), a perceived gap will be observed. A gap between the external or personal requirements and the actual performance is an unsatisfactory situation. An employee can cope with this situation via one or a combination of three different routes.

Employees that follow the first route continue to invest effort or increase their effort to eventually meet the requirements and close the perceived gap. Whether they are inclined to do so depends on their motivation to perform the task and meet the requirements. Following the model (Figure 6), the first route in the model is from the variable “mental effort” to “performance” to “gap in performance”, with the “motivation to meet demands” as a reinforcing factor (+).

**Example** **4.** *Anna’s colleague Sarah has requested that she assist on developing a new product for a highly appreciated client. The success of the product is very important to Sarah. Sarah was a great help to Anna when she started at the company. Anna is strongly motivated to perform well. Although being tired from all the work lately and all the broken nights because of hard work and private situations, she starts right away, putting a lot of effort into the product. After a while she notices that the product that she is working on is not going to meet the requirements. Sarah shows she is a little bit disappointed in the prototype that Anna had made. Anna decides to work harder. She expects Sarah to be very disappointed in her if she does not meet the requirements again. She starts skipping social activities and working overtime becomes routine. Anna is in the “blinded by motivation” loop*.

Following the second route, employees can adjust their demands by adjusting the task load, for example if an employee adjusts their task load by handing over (some) work or if they ask a colleague to help perform the task. Whether the employee is inclined to ask a colleague depends on the expectations of the individual, the consequences of handing over work tasks, and the personal perceived importance attached to the expected consequences. If an employee expects that handing over a task will negatively influence their performance review or the way colleagues will respond and if working long hours is very important for their self-esteem, the employee will be more reluctant to hand over a task. Handing over a task can lower the employee’s self-esteem. On the other hand, if a male employee believes handing over work will give more freedom and that they or the organization care for his wellbeing, the personal experience of the employee will be that handing over a task will not negatively influence his performance review or the way colleagues see him, and that his self-esteem will not be built up by working long hours but by efficient performance, meaning he will be more inclined to hand over work. Following the model (Figure 6), the second route in the model is from the variable “task load” to “demand” to “gap in performance”. Whether the employees are inclined to do so depends on the factors “self-efficacy” (+), “expected consequences of rejecting or accepting tasks and meeting resulting demands” (+), and “importance of work-goals for self-esteem” (+).

**Example** **5.** *Andrew, a senior employee, has been given an extra task by his new manager. The new young manager has recently been assigned because of a re-organization. Andrew always performed well and delivered his work with care, setting a very high standard for himself. Currently, however, besides the stress of the re-organization, his private situation is also putting a lot of constraints on him, reducing the quality of his work. At first, he is not fully aware that it is mainly his personal situation that is demanding extra attention and is the main origin of his lower efficacy. Andrew feels disappointed about his lower quality of work. Additionally, he has the experience that older employees are vulnerable in re-organizations, and not knowing his new manager very well he is reluctant to hand the task back to his manager or to a colleague. His manager or colleagues should be aware and support Andrew before he enters the “pressure to do more” loop*.

A third route in the model is that employees adjust the demand by adjusting their perception or interpretation of the requirements. Employees can also adjust their own personal requirements and lower their own goals, both in order to make it more feasible to meet the requirements. On short notice this route may help the employee, but in the end this might lead to the assignment of less challenging work. Following the model (Figure 6), the third route in the model comes from indirectly lowering the variable “motivation to meet demands” or directly lowering the variable “requirements per task”, resulting in a lower “demands” variable and lower “gap in performance”. As employees lower their “motivation to meet demands”, so does their “perceived capacity for effort” (+), resulting in lower “self-efficacy” (+), and therefore a lower workload.

#### 3.3.3. Impact on the Health of an Employee

The third main process describes the impact on the health of the employee. Work-related stress as defined by WHO “is the response people may have when presented with work demands and pressures that are not matched to their knowledge and abilities, and which challenge their ability to cope” [2]. Work-related stress emerges when employees perceive a gap between requirements and their actual performance, especially when they consider it important to perform (at work) in a certain way and meet the requirements. This can create a state of stress for a prolonged time. Recovery time and appropriate recovery activities can alleviate stress and refill the capacity for effort. Following the model (Figure 6), the “gap in performance” increases, resulting in a higher “mental effort” (+), which then lead to an increase of the “stress” stock (+).

**Example** **6.** *Peter is in charge of integration of two different units within a large company at a new location. The two units differ in service, size, and especially culture. He puts major effort into the optimal information flow, co-creation process for the new-building, and into managing the reluctancy of employees who need to move. The prolonged stress exhausts him, and even after a simple task such as reading a document he needs a 2 h nap to recover. Peter has fallen into the “limits of the body” loop*.

#### 3.3.4. Burnout Symptoms

The fourth main process describes the consequences of prolonged, chronic stress. In the long run, employees become tired and eventually exhausted. Being in a state of stress for a longer period has consequences for their emotional and cognitive functioning. Additionally, depending on the individual health state, chronic stress might negatively influence their physiological functioning. A strong motivation to perform and meet requirements conceals symptoms of exhaustion and impaired cognitive and emotional functioning. In the long run, motivation cannot outrun the symptoms anymore and the “limits of the body” loop starts to govern the model. The employee develops burnout symptoms (work-related fatigue, resulting in exhaustion, cynicism, and reduced efficacy). Following the model (Figure 6), through more “mental effort” and “stress” (+), eventually the “capacity for effort” is degraded by the excessive load. “Cognitive and emotional functions” (+) decrease, adjusting the “performance” to a lower level than what would have been possible. In this case, the situation can be worsened further by a high “motivation to meet demands”, which for some time overrules the “limits to the body” loop. At some point the demands can no longer be met and will start to decline. As “symptoms of exhaustion” decline (−) and one starts learning from (and overcoming) their burnout experience, many people learn to better manage “demands” and “perceptions”. Interestingly, all seven participants reported having learned from the experience and entered the “learning to adjust goals and perception” loop. However, some participants went through multiple cycles of burnout before fully learning how to manage the feedback processes described here.

### 3.4. Simulations of the Effects of Three Different Personas on Devlopment of Burnout and Recovery

The potential for the quantitative SD to visualize the effects of personal perception and behavior on development of chronic stress, burnout, and recovery was evaluated. A quantitative SD model has two distinct purposes: (1) the improvement of decision-making quality by providing a holistic support tool; and (2) to introduce the ability to learn by experimentation. An SD model can function as a boundary object for conversation, investigation, or even formal education. By describing both the structure and behavior, a quantitative SD model can be used to better understand a problem and improve decision-making by experimenting with the system behavior. The user can interact with the model to explore scenarios, including scenarios involving their own past or current behavior.

As an illustration, the potential investigation of burnout in a simulation model is illustrated via three simulations. System behavior was simulated over a 100 week timeframe, which was set by experts based upon the inputs of participants. The simulations are intended as a proof-of-concept, while levels of variables are defined as “low”, “medium”, or “high” and as either static or dynamic, with the latter indicating a change over time. The simulation output of the model is illustrated with three cases, describing three fictitious personas based on the participant responses. To run a simulation, the stock and constant values of the model at t_0_ are set to a specific value. In this way the differences between the personas are quantified. In Table 2, the parameter values that were changed in the model to initiate these scenarios are given in an overview. Note that only a fraction of the parameters is different between the personas, indicating that this a complex system that can display very different behavior based on small differences in the initial condition. The three different personas are described in more detail below.

*Persona 1: Develops severe burnout symptoms but the person learns from this, allowing for a learning effect and recovery*

- This simulation was the closest match with five out of seven scenario case studies that were conducted. The model simulated persona 1, who has the following features:
  ○ Importance of work goals for self-esteem: “medium” and dynamic;
  ○ Expected consequences of (not) meeting demands: “medium” and dynamic;
  ○ Persona 1 will learn from burnout symptoms and their environment and will adjust their future behavior accordingly.

*Persona 2: Develops burnout symptoms but the person does not sufficiently learn from it, leading to a cycle of burnout symptoms*

- This simulation was the closest match with two out of seven scenario case studies that were conducted. The reason why persona 2 does not learn can be multifactorial. A person might have personality traits that result in a specific burnout trajectory or might work in an unhealthy work environment that does not support the learning process. For this simulation, the features of persona 2 are identical to persona 1, except for the following differences:
  ○ Importance of work goals for self-esteem: “high” and dynamic;
  ○ Expected consequences of (not) meeting demands: “high” and dynamic’
  ○ Persona 2 will not learn from burnout symptoms or their environment and consequently will not adjust their “importance of work goals” or “expected consequences of (not) meeting demands” accordingly.

*Persona 3: Is under pressure but the person copes with it*

- This simulation was the closest match with an expert knowledge scenario. Contrary to personas 1 and 2, persona 3 has a static limit on two important variables (as can be seen below). These limits will help persona 3 in not becoming over-invested in their work. These limits might be a personality trait or might be the result of a supportive environment. In addition, if demands do get too high, persona 3 can learn from the experience and their environment and adjust their future behavior”
  ○ Importance of work goals for self-esteem: “medium” but static;
  ○ Expected consequences of (not) meeting demands: “low-medium” but static;
  ○ Persona 3 will learn from burnout symptoms and their environment and will adjust their future behavior accordingly.

**Figure 7:**
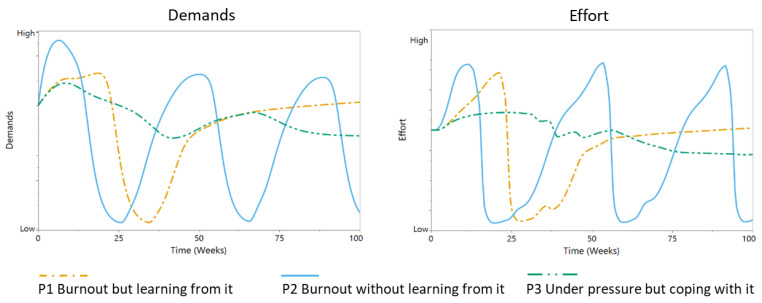
Model behavior patterns for “demands” and “effort”, parameterized for the simulations of personas (P): P1 “burnout but learning from it” (orange dotted line), P2 “burnout without learning from it, (blue line) and P3 “under pressure but coping with it” (green dotted line).

**Figure 8:**
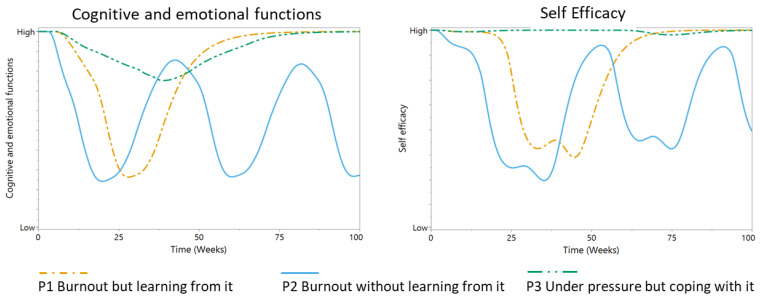
Model behavior patterns for “cognitive and emotional functions” and “self-efficacy”, parameterized for the simulations of different personas (P): ”P1 “burnout but learning from it” (orange dotted line), P2 “burnout without learning from it” (blue line), and P3 “under pressure but coping with it” (green dotted line).

**Figure 9:**
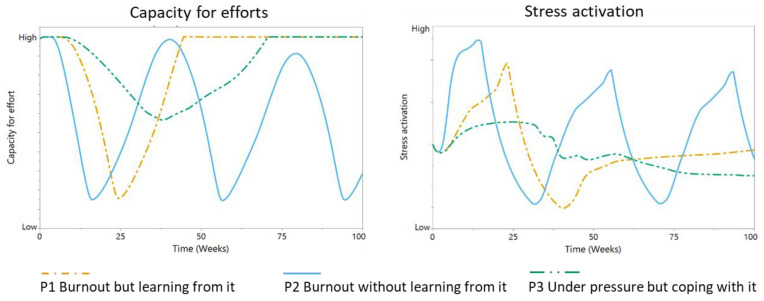
Model behavior patterns for “capacity for effort” and “stress activation”, parameterized for the simulations of personas (P): P1 “burnout but learning from it” (orange dotted line), P2 “burnout without learning from it” (blue line), and P3 “under pressure but coping with it” (green dotted line).

Simulations were run with all three personas starting from the same initial state—they were investing a normal level of effort into their job, their cognitive and emotional functions were as normal, they were confident in their ability to perform their job (“self-efficacy”), they were well rested (“high capacity for effort”), and they showed a healthy level of stress activation. However, the behavior patterns for the three personas are strikingly different (Figure 7, Figure 8 and Figure 9).

Personas 1 and 3 initially show similar development of demands (Figure 7). However, persona 3 is more able to regulate their effort, leading to more sustainable levels of effort, although possibly lower than what could have been achieved. The demands persona 2 must face quickly increase as they are pressured to do more. However, their inability to regulate demands and the effort required to match them with available resources results in a cyclical process of episodes of low and high effort. Initially, persona 1 is also not successful at regulating their demands and effort. After suffering an episode of burnout, they learn to regulate their demands at a sustainable level by adjusting the relevance of their work goals and gaining more insight into the consequences of (not) meeting their demands (Figure 7). We observe similar patterns for the other variables (Figure 8 and Figure 9). Persona 3 is to some degree able to protect their cognitive and emotional functions and succeeds well at limiting their gap in performance, which results in stable levels of self-efficacy (Figure 8).

Persona 1 shows a major decrease in these variables, which can be considered a phase of burnout. Persona 1 can recover from this phase and enter a stable state in which they can meet their demands and retain their cognitive and emotional functions, as well as their sense of self-efficacy. Persona 2, however, is not able to find stability and is trapped in a cycle of burnout episodes. Their behavior is regulated by the limits of their body, not by healthy limits in terms of goals and demands that would help them to achieve sustainable levels of effort.

Persona 1 is initially unable to handle demands, resulting in a peak in stress and a temporarily reduced capacity for effort (Figure 9). However, they recover and learn from the experience, resulting in stable behavior. Persona 3 also needs to adjust to the demands imposed on them, but they succeed in finding a less stressful trajectory that incurs less costs on their capacity for effort. Persona 2 is unable to find a stable level of demands, resulting in episodes of high stress and reduced capacity for effort, followed by peaks.

The results indicate that the steepness of the perceived stress development is an early warning signal for a person heading towards burnout. At the same time, scenario analyses indicated that the steepness in stress is the result of not being able to react appropriately, either due to individual characteristics or a nonsupportive environment. This suggests that burnout prevention programs focusing on susceptible personas alone might not be effective, and a positive work environment with shared awareness of early warning signals and efficient supportive actions taken for the individual’s context (colleagues, managers, family) is crucial to promote vitality at work.

## 4. Discussion

This study takes a dynamic complex system view of the individual’s perceptions and behavior leading to chronic stress, burnout, and recovery. From a dynamic complex system view, we conceptualized burnout as the outcome of a system deteriorating over time, and as such a process that may be reversed earlier when the complexity of the problem is better understood. The aim of this paper is to describe the applicability of a combined modeling approach to develop a SD simulation model of burnout development and recovery, with the ultimate aim of using it as an intervention tool for educational and awareness purposes. We have presented a modeling approach that visualizes both the structure and the behavior of burnout. At first, an expert-informed conceptual model was built, confirmed, and supported by literature. Thereafter, the need for additional longitudinal case data became apparent and an experimental technique was developed asking participants to “quantify” and to “draw their own dynamics” to collect retrospective personal data. The combination of both expert knowledge and literature, together with personal scenarios, was used to develop a proof-of-concept SD simulation model to visualize different burnout development and recovery trajectories.

With an increasing burden on society from mental- and lifestyle-related health problems, it is essential to develop ways that can support effective behavior change and promote vitality [6]. Especially in today’s complex and uncertain work environment, for instance due to increasing innovative technology development, cultural diversity, and competitiveness, there is a need for a better quantitative and qualitative understanding of the interrelationships between the variables leading towards chronic stress and burnout. Health prevention program managers, decision makers, and employees need new insights into effective strategies and tools that can prevent burnout prior to using real actions to prevent aversion, unwanted burden, and unnecessary costs. Successful prevention and intervention programs involve employees in decision-making and enhance their job control and social support, while the elimination of stressors explains the effectiveness of the prevention and intervention programs [42]. SD has proven to be effective in modeling and analyzing business and healthcare systems characterized by dynamic complexity and uncertainty, as well as experimenting with scenario simulation to design and evaluate interventions for performance management [43,54,57,66,67,68]. Effective behavioral and cultural change, however, can only be achieved when all crucial elements supporting an effective change are available, including a shared vision, shared mission, clear goals and outcomes, shared responsibility, appropriate skills, supporting environment, and appropriate resources [69].

In this paper, we demonstrated how a SD model can be developed and used to visualize the structure and behavior of variables that influence burnout, such as the demands, resources, perceptions, and behavior. We illustrated how SD can be used to describe the feedback mechanisms that underlie very personal and context-related health problems such as burnout. The validity of the model can only be viewed with regards to the purpose of illustrating the impacts of the feedback mechanisms on the outcomes over time [70,71]. The ultimate purpose of this model is to create new insights and shared awareness by informing (potential) victims of burnout, providing a visual summary to deepen understanding on how burnout develops and what one might do about it.

Establishing the usefulness of the model starts with validation of the model structure. The question must be asked whether there is enough confidence that the model describes the structure of variables and relations that lead to burnout. The validity of the model structure needs to be tested directly by confronting the model with knowledge of the scenarios leading towards burnout in real life. The current version of the model is based on the synthesis of existing theoretical models that describe burnout and other relevant elements. The synthesis was supported and confirmed by experts. In addition, an in-depth study of 7 personal burnout and recovery scenarios was conducted. With these personal case data, the relationships between the variables and the systemic behavior of the system were improved. This is only a starting point; the model will benefit both from the continuously evolving theory on burnout, as well as more personal case studies from different work contexts. Critical review will remain essential. To this end, several simulations of fictitious cases based on the 7 case studies in a Western working environment were performed and presented. These simulations qualitatively matched the behavior over time that was described by the burnout victims. Although the hard evidence is preliminary, we conclude that the SD model produces “the right behavior for the right reasons” [52].

System structuring and system dynamics have previously been shown to be effective for supporting new insights and behavioral change [56,72,73]. Through simulations of “what if” scenarios, employees, managers, and care providers can discuss hypotheses and possible outcomes for both current behavior and interventions. This can help organizations to develop prevention and intervention programs aimed at workplace stress and burnout.

A model by itself, however, will not change the deeply held assumptions, routines, communication patterns, and organizational properties that lead to burnout. This will require the model to be made accessible to people. From a technical perspective, the bridge between the model and the user can be crossed by providing outputs in graphical user interfaces, including game elements, and by collecting inputs via apps or wearables. In addition, action research could prove helpful in designing the process with which people can be best assisted in developing a systemic perspective on burnout. Shared decision-making in healthcare is gaining increasing interests. Elwyn et al. [74] defined shared decision-making is a process in which decisions are shared between decision makers (team managers, professionals) and clients (employees, patients), informed by the best evidence available and weighted according to the specific characteristics and (perceived) values of all stakeholders involved. This research, therefore, advocates that effective information about behavior and interrelationships between individuals and contexts over time from a complex system view is essential to help optimize and prioritize activities that support vitality programs to prevent chronic lifestyle-related health problems.

### 4.1. Limitations

This study has several limitations that must be considered. First, only a small number of participants who were willing to share their personal experience and perspectives on the dynamics between work and private determinants could be recruited within the research project timeline. Furthermore, the participants were all desk researchers working at the same project-driven applied research institute within the Netherlands. It is expected that both work-, sector-, culture-, and socioeconomic-related status differences may influence the dynamics between the variables to a certain extent, meaning further research is necessary. The current work should, therefore, be seen as a proof-of-study for developing a SD simulation model of development of burnout and recovery. The current work needs improvement. Improvements can be made to the accuracy of the model, its ability to simulate different personas, and the scope of the variables included in the simulation. The usefulness of the model might further be improved by including more aspects of the context in which an individual works and lives. For example, an investigation of the roles of communication, team managers, social environments, diversity, different cultural worldviews, and socioeconomic status could be included. Another limitation is that we have focused on the European work environment; therefore, similar research needs to be conducted in other cultures.

### 4.2. Comparison to Previous SD Assessments of Burnout

Compared to existing SD studies of burnout, the current study integrates a systemic worldview with the existing body of literature on burnout. In addition, this study used graphs-over-time from multiple participants to calibrate and validate the model. The variables and feedback dynamics in the current model share some similarities with other system dynamics models. Jetha et al. [45] conceptualized the dynamics of workplace stress experienced by nursing aides and described the relationships between their job resources, job demands, and stress. The model presented here is a considerable extension, which includes numerous other variables and feedback loops describing the onset and recovery of burnout. Homer was the first to describe some key dynamics of employee burnout, including the cyclical nature of burnout if no learning occurs [54]. Homer, however, based his SD model solely on his own experience, whereas Jetha et al. based their model on the inputs of several experts. The work by Wu et al., having a different focus, used SD modeling to explore the impacts of work–family conflict, mainly on the motivation and performance of the employee [55].

## 5. Conclusions

In the present study, we conceptualized burnout as the outcome of complex and dynamic interactions between various variables in so-called feedback loops. These interactions lead to an unhealthy state, the development of which may be reversed early when the feedback mechanisms involved are better understood by the person and the environment. The participants in this study who have experienced burnout and have recovered all seem to possess this understanding.

A new approach in the field of burnout was successfully applied to model the development of chronic stress, burnout, and recovery. This study demonstrated that a SD model could be developed based upon expert knowledge, literature review, and retrospective longitudinal data. In addition, the case data could be approximated by a stock-and-flow simulation model. The model was able to simulate realistic long-term patterns of chronic stress and emergence of burnout for persons working in a project-driven research institute in the Netherlands.

A major contribution of is this study is the synthesis of many different feedback loops and their interactions underlying personal and context-related burnout relationships into one dynamic model. The current study integrates an important part of the existing body of knowledge into one systemic perspective. The simulations suggest that employees being able to recognize, monitor, and steer these feedback mechanisms, either on their own or with their colleagues, could be crucial in preventing chronic stress and burnout. We advocate that burnout prevention programs need to focus on a positive work environment with shared knowledge of the feedback mechanism involved. Shared knowledge enables shared responsibility for taking actions on early warning signals of chronic stress development. Additional research is needed to identify the specific strength of the relations in the model and to include more personal scenarios in order to further validate and extend the current (European-based) model. For other regions, cultures, and different work contexts, it may be interesting to develop specific versions of the model.

In conclusion, the results indicate that simulation of realistic personal scenarios of burnout is feasible. This proof-of-concept SD simulation model of burnout and recovery can be further developed into a tool that can be used as part of organizational interventions or as part of organizational change programs for promotion of health and wellbeing at work, creating a shared and systemic view of the dynamics leading to burnout. Shared decision-making between decision makers (team managers, professionals) and clients (employees, patients) in intervention strategies is gaining increasing interest. Accessible, easy-to-understand, and effective information on the different determinants and the relationships involved could be helpful in designing the process with which people can be best assisted in developing a systemic perspective on burnout.

## Figures and Tables

**Table 1:** The existing theoretical models included in the system dynamics (SD) burnout model, their relevance to the SD model, and references.

Description	Relevance to SD Burnout Model	References Used
**The revised job demands–resources (JD-R) model:** Model with two key proposed processes: (1) the energetic process (leading to exhaustion when a person is faced with high job demands and insufficient recovery; and (2) the motivational process, describing how having sufficient resources to meet demands can lead to engagement.	Understanding, parameterizing, and defining the dynamic interactions between job demands, job resources, and engagement. For example, the ‘regulating demands’ loop.	[22,23,26,27,28,63]
**The compensatory control model and the effort–recovery model:** These models describe the coping–control element of the burnout system. If the perceived effort is higher than the current effort, balance can be restored via: (1) increase of effort (resulting in physiological costs and higher load); or (2) maintaining current demands (performance stays equal but lower effort—costs will be saved).	Understanding, parameterizing, and defining the dynamic interactions between demands, effort, and recovery. For example, the ‘regulating effort’ loop.	[20,29,30,31]
**The model of daily recovery from work and model of work, recovery, and health:** These models describe the recovery element of the burnout system. After an effort is made, recovery is necessary through resting or in other forms. Short-term effects of fatigue are related to an individual’s psychological and energetic state during the workday. Long-term effects of fatigue are related to lower work performance, absence, and chronic physiological and psychological health impairments. Overwork and after-hours stress can hamper recovery; a state of sustained activation gradually exhausts the person and can lead to burnout. Recovery from long-term effects (“allostatic load”) is much more difficult and takes a prolonged amount of time.	Understanding, parameterizing, and defining the role of capacity for effort, recovery, and the effects of fatigue. For example, the ‘limits of the body’ loop.	[32,33,34]
**The effort–reward imbalance (ERI) model** Model of the importance of the imbalance of effort and reward, based on the principle of “social reciprocity”. Assumption: if effort is invested, a person expects a reward. If high effort is invested while low rewards are received, the model predicts that negative emotions and a sustained stress response will result. An imbalance might be maintained by a person if they (1) are left with no options; (2) temporarily accept the situation for strategic reasons; or (3) if a motivational pattern of “over-commitment” exists.	Understanding, parameterizing, and defining the dynamic interactions between effort, elements of reward, and motivation. For example, the ‘blinded by motivation’ loop.	[35,36]
**The challenge–hindrance occupational stress model, role of appraisal, and threats** Demands are appraised to determine the appropriate stress and effort response. A distinction is made between job hindrances, challenges, and threats. Job challenges are those demands for which a person feels are aligned with their goals and for which sufficient resources are available. Job hindrances are aspects of one’s job that hinder a person from obtaining desired outcomes. Threats are those aspects that are appraised as posing a risk to the person’s performance or wellbeing.	Understanding, parameterizing, and defining the appraisal process of job demands and behavioral response. For example, the dynamics surrounding stress.	[37,38,64]

**Table 2:** Parameterization of the scenario runs, simulating the effect of three different personas (P) on the development of burnout and recovery. Runs are performed by a combination of parameter changes in policy values, initial states, and switches.

Simulation of Persona (P)	Maximum Expected Consequences	Maximum Work Goal Relevance	Work Goal Relevance	Expected Conse-Quences	SWITCH Allow Expected Consequences to Change	SWITCH Allow Work Goal Relevance to Change	SWITCH Learning
P1: Burnout and learning from it	1	1	0.6	0.6	1	1	1
P2: Burnout and no learning	1	1	0.9	0.9	0	0	0
P3: Under pressure but coping	0.5	0.7	0.6	0.6	1	1	1
